# Aggregation-Tuned Charge Transport and Threshold Voltage Modulation in Poly(3-hexylthiophene) Field-Effect Transistors

**DOI:** 10.3390/ma19020279

**Published:** 2026-01-09

**Authors:** Byoungnam Park

**Affiliations:** Department of Materials Science and Engineering, Hongik University, 72-1, Sangsu-dong, Mapo-gu, Seoul 04066, Republic of Korea; metalpbn@hongik.ac.kr

**Keywords:** P3HT, sonication, aggregate, mobility, threshold voltage, FET

## Abstract

In this report, a thickness-driven, aggregation–structure–transport optimum in sonicated poly(3-hexylthiophene) (P3HT) FETs was investigated. Mobility peaks at ~10–20 nm, coincident with a minimum in the photoluminescence (PL) vibronic ratio $I_{0-0}/I_{0-1}$ (strong *H*-aggregate interchain coupling) and X-ray diffraction sharpening of the (100) lamellar peak with slightly reduced *d*-spacing, indicate tighter π–π stacking and larger crystalline coherence. Absorption analysis (Spano model) is consistent with this enhanced interchain order. The mobility maximum arises from an optimal balance: *J*-aggregate–like intrachain planarity supports along-chain transport, while *H*-aggregates provide interchain connectivity for efficient hopping. Below this thickness, insufficient interchain coupling limits transport; above it, over-aggregation and disorder introduce traps and weaken gate control. The sharp rise in threshold voltage beyond the critical thickness indicates more trap states or fixed charges forming within the film bulk. As a result, a larger gate bias is needed to deplete the channel (remove excess holes) and switch the device off. These results show that electrical gating can be tuned via solution processing (sonication) and film thickness—guiding the design of P3HT devices for photovoltaics and sensing.

## 1. Introduction

Conjugated polymers have emerged as a cornerstone material class for a new generation of electronic devices, prized for their solution-processability, mechanical flexibility, and low fabrication cost [1,2,3,4]. These attributes have enabled significant progress in diverse applications, including organic field-effect transistors, organic photovoltaics, and chemical sensors [5]. Among the vast library of available materials, poly(3-hexylthiophene) (P3HT) has been established as the archetypal semiconducting polymer, serving as a benchmark system for fundamental studies due to its robust processability, relatively high charge carrier mobility, and well-documented self-assembly characteristics [6,7,8,9].

It is now unequivocally understood that the performance of devices based on these materials is not merely a function of the polymer’s intrinsic chemical structure but is critically governed by its solid-state nanoscale morphology [1]. The arrangement of polymer chains, the size and connectivity of crystalline domains, and the nature of molecular packing directly facilitate or impede charge transport, thereby dictating key device metrics such as charge carrier mobility, current on/off ratio, and quantum efficiency [10,11]. Consequently, the rational control of this morphology through processing has become a paramount objective in the field of organic electronics. This challenge requires moving beyond a simplistic binary view of “crystalline” versus “amorphous” regions toward a more sophisticated understanding of the specific molecular packing motifs that constitute the ordered domains.

In this context, the concepts of *H*- and *J*-aggregation, originally developed to describe excitonic coupling in molecular dyes, provide a powerful framework for analyzing the structure of conjugated polymer films [12,13]. These aggregate types correspond to distinct molecular arrangements that create unique optical and electronic properties. Controlling the balance between *H*- and *J*-aggregates allows the material’s function to be tuned precisely. Film thickness is a critical processing parameter that strongly influences molecular self-assembly [13,14]. Although thicker films may improve light absorption or conductivity, the connection between thickness and performance is complex and often non-linear, as thickness variations can introduce structural defects or alter aggregation behavior [15]. Consequently, investigating the thickness-dependent formation of *H*- and J-aggregates is essential for developing design principles to optimize device performance. Such a study elevates the discussion from a materials processing problem to a fundamental inquiry into the interplay between dimensional confinement and excitonic physics in dictating charge transport.

To understand the structure-property relationships in P3HT, it is essential to define the distinct roles of *H*- and *J*-aggregates [15,16,17,18]. These classifications describe the nature of excitonic coupling between chromophoric units, which in a polymer context, relates to interactions both along a single chain and between adjacent chains. *H*-aggregates arise from through-space Coulombic interactions between polymer chains arranged in a cofacial, side-by-side configuration, characteristic of π–π stacking. This arrangement results in a positive excitonic coupling, which places the optically allowed excited state at the top of the exciton band. Spectroscopically, this manifests as a characteristic blue-shift in the absorption spectrum relative to the isolated chromophore and a suppression of the lowest-energy (0-0) vibronic transition in both absorption and PL spectra. *J*-aggregates, in the context of a single polymer chain, are a consequence of through-bond electronic interactions that favor a planar, delocalized backbone conformation. This leads to an effective negative excitonic coupling, placing the optically allowed state at the bottom of the exciton band. The spectroscopic signature of *J*-aggregate behavior is a red-shift in the absorption spectrum and a pronounced enhancement in the 0-0 vibronic peak.

The seminal theoretical work by Spano and colleagues established that semicrystalline P3HT films are best described as a composite “*HJ*-aggregate” system, where these two effects coexist and compete [19,20]. Within this model, the planar backbone of an individual chain promotes *J*-like character, while the intermolecular π–π stacking introduces *H*-like coupling. The final optoelectronic properties of the film are determined by the delicate balance between the strength of intrachain (*J*-promoting) and interchain (*H*-promoting) interactions. This balance can be quantitatively assessed by analyzing the intensity ratio of the 0-0 to 0-1 vibronic peaks in the absorption spectrum ($A_{0-0}/A_{0-1}$), which serves as a powerful experimental probe of the dominant coupling mechanism.

Crucially, these distinct aggregate types are directly linked to the fundamental mechanisms of charge transport. High *J*-aggregate character is a hallmark of superior intrachain order and backbone planarity, which minimizes torsional defects and maximizes π–π orbital overlap along the chain. This creates a highly efficient, quasi-one-dimensional pathway for intrachain charge transport [21]. Conversely, *H*-aggregate character signifies close and ordered π–π stacking between adjacent chains, providing the essential electronic coupling for charges to hop from one chain to another. This interchain hopping is vital for creating a continuous, percolating transport network that connects the high-mobility intrachain pathways and allows charges to navigate around defects [21].

Optimal device performance, therefore, depends on achieving a synergy between these two transport mechanisms. While the planar backbones associated with *J*-aggregates provide rapid, quasi-1D transport “highways,” the realization of a functional 2D device channel necessitates pathways for charges to move between these chains. This interchain hopping, mediated by *H*-aggregates, represents a kinetically slower step and often constitutes a critical transport bottleneck, especially given that intrachain mobility in P3HT can be orders of magnitude higher than interchain mobility [11]. The central challenge in morphology engineering is thus to establish sufficient interchain connectivity to form a robust network without excessively disrupting the highly desirable planar order of the individual chains.

A variety of solution-based processing strategies have been developed to manipulate the nanoscale morphology of P3HT films [21,22,23]. These include the judicious selection of solvents with different boiling points to control evaporation rates, as well as post-deposition thermal or solvent vapor annealing to promote molecular rearrangement and enhance crystallinity [24,25]. Among pre-deposition techniques, sonication of the polymer solution has proven to be a particularly potent method for improving device performance. The application of ultrasonic energy disentangles polymer chains in solution, breaking up kinetically trapped random coils and lowering the energetic barrier for the formation of ordered nuclei during film casting. This process has been shown to yield improvements in organic field effect transistor (OFET) mobility by one to two orders of magnitude. However, recent evidence suggests that this mobility enhancement is correlated with the preferential formation of *H*-aggregates, which promote interchain connectivity. This raises a critical question: does this *H*-aggregate promotion come at the expense of the highly efficient, *J*-aggregate-like intrachain transport pathways? If so, sonication alone may not be sufficient to achieve an optimal morphology, instead requiring a complementary processing parameter to fine-tune the final structural balance.

Film thickness represents another critical, yet incompletely understood, control parameter. Previous studies have consistently shown that OFET mobility is strongly dependent on the thickness of the P3HT active layer, often exhibiting an initial increase followed by saturation or a decrease for thicker films [26,27,28]. This non-monotonic behavior is generally attributed to a transition from a transport regime dominated by the 2D channel at the semiconductor-dielectric interface to one governed by 3D bulk conductance. In thicker films, charge transport can be degraded by an increase in bulk traps and a weakening of the gate field’s ability to modulate the entire channel volume. While these general trends are recognized, a detailed mechanistic understanding that directly links film thickness to the underlying *H*/*J* aggregate balance remains elusive.

This reveals a significant gap in the current literature. The *H*-aggregate-promoting nature of sonication and the morphology-modulating effect of film thickness have been investigated largely in isolation. Their synergistic interplay—how the pre-ordered state induced by sonication evolves under the dimensional constraints imposed by film thickness—has not been systematically explored. This leaves a critical void in the ability to rationally design processing protocols for high-mobility P3HT devices. It is plausible that sonication creates a solution state primed for strong *H*-aggregation, and film thickness then acts as a crucial second tuning knob. The kinetic and thermodynamic environment during solvent evaporation, which is heavily influenced by film thickness, could mediate how this pre-aggregation translates into the final solid-state structure, potentially allowing for the preservation of *J*-like planarity in ultra-thin films while promoting excessive *H*-aggregation and disorder in thicker films.

In this work, we systematically investigate the interplay between pre-deposition sonication and film thickness to rationally control the aggregation, structure, and charge transport properties of P3HT FETs. We identify a distinct charge carrier mobility maximum at a film thickness of approximately 10–20 nm. Spectroscopic and structural analyses reveal that this performance peak coincides with a morphology that optimally balances two competing requirements for efficient charge transport. At this critical thickness, the P3HT film exhibits strong *J*-aggregate-like character, indicative of high intrachain planarity that supports rapid charge transport along the polymer backbones. Simultaneously, it maintains sufficient *H*-aggregate-like interchain coupling to provide the necessary connectivity for charges to hop between chains, thus forming a continuous and efficient transport network. This study provides a clear design rule for optimizing P3HT-based devices and demonstrates a powerful strategy for tuning electrical properties through the synergistic combination of solution-state pre-processing and solid-state dimensional control.

## 2. Materials and Methods

### 2.1. P3HT Film Formation and Four-Contact FET Fabrication

Highly regioregular P3HT (>96%, Sigma Aldrich, WI, USA) was used as received. Solutions of P3HT in chloroform with varying concentrations from 0.5 mg/mL to 10 mg/mL were prepared for film deposition by spin-coating at 1500 rpm for 30 s onto the SiO_2_ gate dielectric layer for four-contact field effect transistor (FET) fabrication. To induce a more ordered molecular arrangement within the P3HT films—essential for enhanced electronic performance—the solutions were sonicated at 50 W and 40 kHz in a chilled water bath for 10 min prior to spin-coating.

To evaluate charge transport in P3HT layers near the SiO_2_ interface while eliminating the influence of metal/semiconductor contact resistance, a bottom-contact FET configuration equipped with two voltage probes between the source and drain electrodes was employed. The device featured photolithographically patterned source and drain electrodes composed of an 80 nm thick gold (Au) layer deposited over a 3 nm titanium (Ti) adhesion layer on a 200 nm SiO_2_ gate dielectric. A heavily doped silicon substrate served as the global gate electrode. To prevent oxidation of both pristine and sonicated P3HT films, a protective encapsulation procedure was applied. Each film was sealed beneath a glass slide using a carefully applied adhesive tape, ensuring an airtight and durable barrier that preserved the film’s integrity during electrical characterization.

### 2.2. Analysis of Four-Contact Effective Mobility and Threshold Voltage

FETs were evaluated in the linear operation regime, where a small drain voltage of −3 V was applied. The effective four-contact field-effect mobility ($\mu_{eff}$), extracted from gated sheet conductance measurements, is distinguished from the conventional two-contact mobility (*μ*) obtained from standard transistor transfer curves. During measurement, the voltage probes ($V_{1}$ and $V_{2}$) between source and drain electrodes monitored the potential drop across the channel under an applied gate voltage exceeding the threshold voltage, which induced mobile charge carriers in the channel. The sheet conductance, $\sigma_{sq}$, was calculated according to Equation (1):

(1) $$\sigma_{sq}=\frac{I_{D}\cdot d_{12}}{W\cdot V_{12}}$$

where $d_{12}$ and $V_{12}$ are the probe spacing and potential difference between $V_{1}$ and $V_{2}$, respectively, *W* is the channel width, and $I_{D}$ is the drain current. From the plot of sheet conductance as a function of gate voltage, the four-contact effective mobility and threshold voltage were extracted using Equation (2):

(2) $$\sigma_{sq}=\;\mu_{eff}C_{i}\;\left(V_{G}\;-\;V_{T}\right)$$

This relation holds in the linear regime of transistor operation, where the induced charge carriers are mobile.

The threshold voltage ($V_{T}$) represents the minimum gate voltage required to induce sufficient mobile charge carriers in the channel to initiate conduction. Once this voltage is exceeded, the drain current increases linearly. In practice, *V_T_* is determined from the sheet conductance as a function of gate voltage ($\sigma_{sq}$ vs. $V_{G}$) in the linear regime. The threshold voltage corresponds to the point where the extrapolated linear portion of the curve intersects the sheet conductance axis ($\sigma_{sq}$ = 0), marking the transition from the non-conducting (off) state to the active conduction state of the FET.

### 2.3. Characterization of Electrical, Optical, and Structural Properties of P3HT Films

The FET characteristics were evaluated using an HP4145B semiconductor parameter analyzer, with all electrical measurements performed in an argon-filled glove box to prevent oxidation and ensure measurement stability. The optical properties, including absorption and PL, were characterized using UV–Vis absorption and PL spectroscopy. For PL analysis, the P3HT films were excited with a 450 nm light source, and the emission spectra were collected in the 650–725 nm range, corresponding to the characteristic luminescence region of P3HT. The degree of molecular ordering in the films was further confirmed through out-of-plane X-ray diffraction (XRD) measurements.

## 3. Results and Discussion

**Figure 1:**
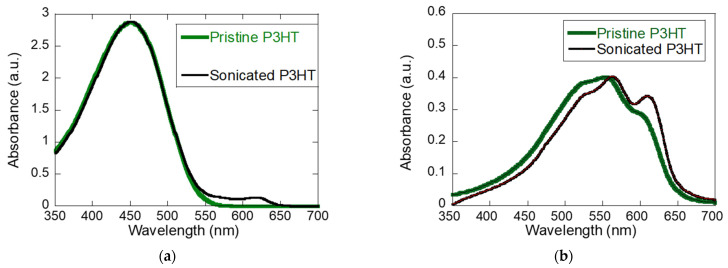
(**a**) UV–vis absorbance spectra of pristine P3HT (green) and sonicated P3HT (black) in solution (5 mg/mL). The spectra show overlapping features, indicating that sonication does not significantly alter the main absorption characteristics in solution. (**b**) UV–vis absorbance spectra of thin films (~30 nm) prepared from pristine P3HT (green) and sonicated P3HT (black).

The absorption spectra in Figure 1 highlights the impact of sonication on the aggregation behavior of P3HT. In solution (Figure 1a), pristine P3HT shows a single broad peak near 450 nm, characteristic of disordered chains with minimal interchain interaction. In contrast, sonicated P3HT exhibits additional vibronic shoulders around 600–650 nm, indicating the onset of π–π stacking and *H*-type aggregation induced by vibration [13]. When cast into films (Figure 1b), both pristine and sonicated P3HT develop vibronic structure, but the features are sharper and more pronounced in the sonicated film, along with a red-shifted absorption edge. These results demonstrate that pre-aggregation in solution is preserved in the solid state, yielding enhanced crystallinity, reduced torsional disorder, and extended conjugation length, which are favorable for charge transport.

**Figure 2:**
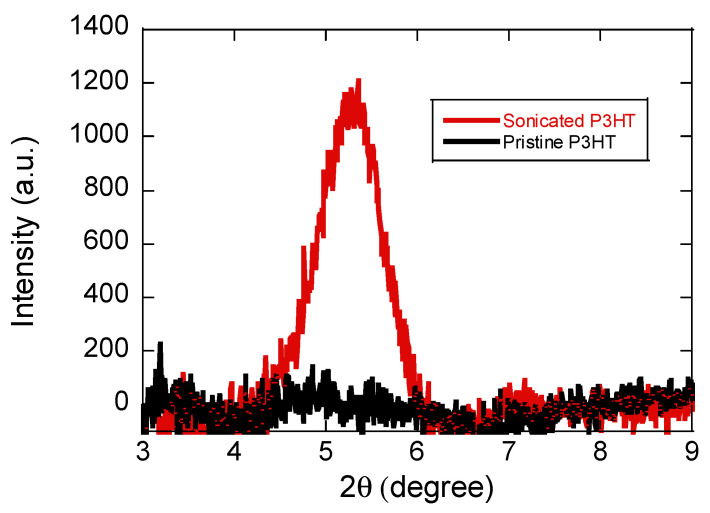
XRD patterns of pristine P3HT (black) and sonicated P3HT (red) thin films (~20 nm).

XRD measurements (Figure 2) confirm enhanced crystallinity in sonicated P3HT compared to pristine films. The sonicated P3HT exhibits a sharp (100) lamellar peak with a *d*-spacing of 16.5 Å, indicating stronger interchain interactions from vibration-induced aggregation. Sonication treatment promotes backbone planarization and extended conjugation length, and this ordered arrangement in solution is retained in the solid state. Previous studies also report that increased aggregation yields more distinct nanofibrillar morphology with improved crystallinity, consistent with findings here [29].

As clearly observed in the atomic force microscopy analysis in the Supporting Information, the pristine P3HT film (Figure S1a) exhibits a relatively featureless and amorphous surface morphology with a lower root-mean-square (RMS) roughness of 0.8 nm. In contrast, the sonicated P3HT film (Figure S1b) displays a distinct nodular texture with increased surface rugosity (RMS = 2.5 nm). This morphological evolution provides direct visual evidence of the enhanced aggregation. The formation of these distinct, interconnected domains in the sonicated film is consistent with the presence of semicrystalline *H*-aggregates and the sharper (100) diffraction peaks observed in the XRD data. The increased rugosity in the sonicated films is a characteristic signature of this crystalline ordering, which facilitates the improved interchain charge transport reported in the electrical characterization.

**Figure 3:**
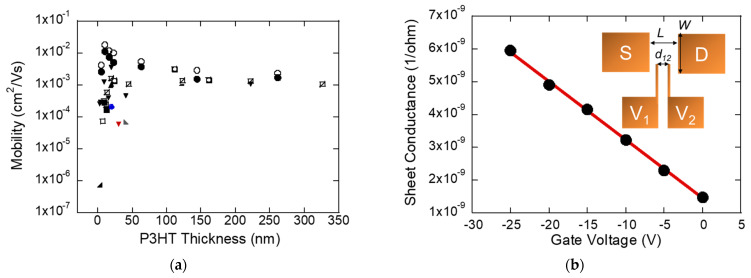
(**a**) Mobility as a function of P3HT film thickness measured using a four-contact device geometry. The data illustrates the dependence of charge carrier mobility on film thickness over a wide range. Each symbol type assigns data to a specific fabrication batch processed on a separate day, illustrating the consistency of device performance across multiple independent trials. (**b**) Sheet conductance as a function of gate voltage plotted on a semilogarithmic scale, with a linear fit (red line) and the corresponding fit equation and R value. ($\mu_{eff}$ = 0.02 cm^2^/Vs, *L* = 200 µm, *W* = 2.8 mm, $d_{12}$ = 80 µm. Mobility is 0.01 cm^2^/Vs.) The inset schematic shows the configuration of source (S), drain (D), and voltage probes ($V_{1}$, $V_{2}$).

For sonicated P3HT exhibiting a more ordered structure, thickness-dependent charge transport was measured using four-contact FETs (inset), which remove the influence of metal/semiconductor contact resistance. In Figure 3a, the effective mobility ($\mu_{eff}$) is plotted versus P3HT thickness; each data series corresponds to a single fabrication batch. The four-contact effective mobility was extracted from the sheet conductance ($\sigma_{sq}$) versus gate voltage characteristics of a representative device at the peak-mobility condition (Figure 3b). The $\sigma_{sq}$–$V_{G}$ relation is distinctly linear, consistent with operation in the linear regime; the slope yields the field-effect effective mobility, while the x-intercept provides the threshold voltage. The excellent linear fit (*R*^2^ ≈ 0.99) underscores the robustness of this extraction and the high transport quality achieved at the optimized thickness.

For ultrathin films (<8 nm), the mobility is severely limited—spanning ~10^−6^ to 10^−3^ cm^2^/V·s—because, at the early growth stage, polymer chains are poorly connected and lack sufficient crystalline order to support efficient transport. As thickness increases, percolation pathways form with additional P3HT, improving connectivity and mobility. As the concentration increases, the mobility rises sharply, indicating the formation of percolative transport pathways enabled by enhanced interchain ordering and aggregation. However, once the thickness exceeds approximately 15–20 nm, the mobility stops increasing beyond its peak value of 0.02 cm^2^/V·s and instead either plateaus or fluctuates around a stable value of about 10^−3^ cm^2^/V·s. This behavior demonstrates that an optimum thickness exists where structural ordering and aggregation effects maximize charge transport, while further thickness provides diminishing or even negative returns due to disorder and additional trapping sites. Taken together, Figure 3a,b highlight that thickness-dependent aggregation strongly influences charge transport, with sonication-induced ordering enabling an optimum thickness for maximized mobility, which is directly reflected in the sheet conductance–gate voltage characteristics, excluding the contact effects.

**Figure 4:**
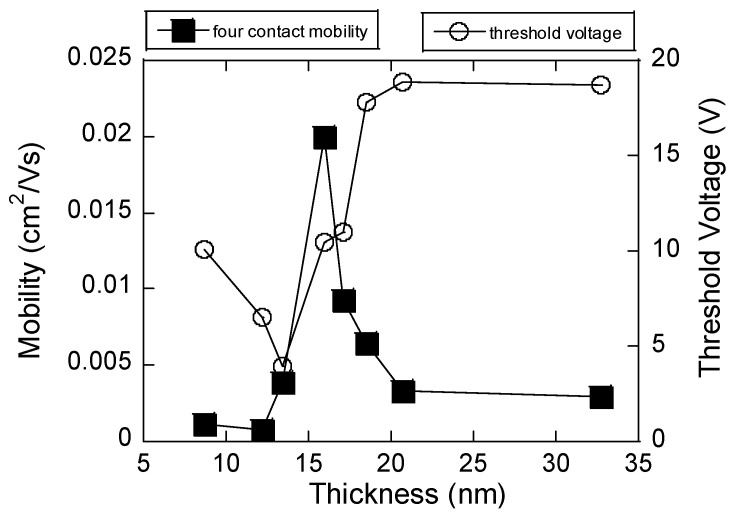
Dependence of four-contact effective field-effect mobility (left *y*-axis, black squares) and threshold voltage (right *y*-axis, open circles) on P3HT film thickness (*x*-axis).

Figure 4 presents the variation in four-contact effective mobility (solid squares) and threshold voltage (open circles) as a function of P3HT film thickness. The mobility shows a non-monotonic dependence, first remaining very low in thinner films (<12 nm), then rising sharply to a maximum value of approximately 0.02 cm^2^/V·s around 15–16 nm. This peak corresponds to an optimum thickness at which the polymer chains exhibit the most favorable molecular ordering and interchain coupling, consistent with strong aggregation-induced crystallinity. Beyond this optimum, the mobility rapidly decreases and stabilizes at lower values, indicating that additional thickness introduces structural disorder, trap states, or exciton quenching that hinder efficient charge transport. In contrast, the threshold voltage displays an opposite trend. At very thin films, the threshold voltage fluctuates at relatively low values, reflecting incomplete accumulation of mobile carriers. As the film thickness increases, the threshold voltage rises steadily and saturates around 18–20 V for films thicker than 20 nm.

It should be noted that post-deposition thermal annealing was not performed in this study. However, the drying process in the inert atmosphere was observed to influence the device characteristics. It is noted that a stabilization of the threshold voltage ($V_{T}$) was accompanied by a slight positive shift (~2 V) after complete drying. This behavior suggests a mild doping effect or structural relaxation during solvent removal, which increases the accumulation of mobile holes at zero bias, thereby influencing the off-state current and the final position of $V_{T}$.

In previous work, we showed that sonicated P3HT exhibits a clear transport optimum: vibration-induced aggregation enhances crystallinity and produces a pronounced mobility peak, whereas pristine P3HT displays a more monotonic mobility saturation during in situ channel formation [30]. The stronger anisotropy of chains in the sonicated solution—evident from the absorption features in Figure 1—also correlates with a more abrupt post-peak current decline during drying. Mechanistically, the transient current peak arises from a balance between two competing processes. Early in solvent evaporation, aggregation of disentangled P3HT chains improves interchain connectivity and raises the current. As solidification proceeds and the solution concentration increases, chain immobilization and emerging microstructural constraints suppress transport, causing the current to fall. Thus, there exists an optimal P3HT concentration window during evaporation at which electronically percolating pathways are most efficiently formed, and this window is extended and amplified by sonication-driven aggregation.

**Figure 5:**
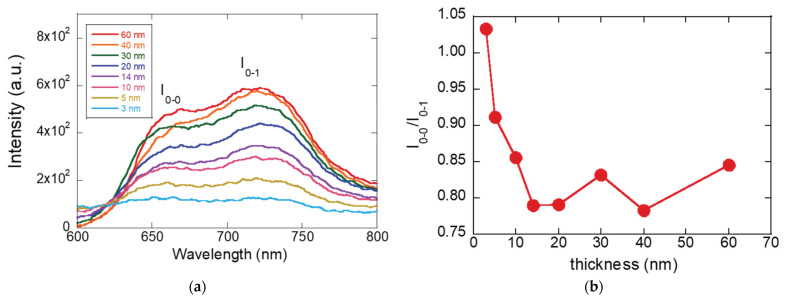
(**a**) PL spectra of P3HT films with varying thicknesses (3–60 nm) showing intensity as a function of wavelength. The emission intensity increases with film thickness, and spectral features evolve with thickness. (**b**) Plot of the $I_{0-0}/I_{0-1}$ vibronic peak intensity ratio versus P3HT film thickness, illustrating changes in excitonic coupling and aggregate formation with increasing thickness.

In this context, we probed how *H*- and *J*-aggregate formation influences charge transport by performing thickness-dependent PL and absorption measurements. Figure 5a presents the PL spectra of sonicated P3HT films with varying thicknesses prepared from different concentrations. The vibronic ratio analysis in Figure 5b identifies a pronounced minimum in $I_{0-0}$/$I_{0-1}$ around 10–20 nm. This minimum indicates strong *H*-aggregate formation, where interchain coupling is maximized and exciton quenching becomes most efficient. Notably, this thickness regime closely matches the mobility peak observed in Figure 3 and Figure 4, confirming that enhanced charge transport arises from the same aggregation-driven ordering that suppresses luminescence. An increase in the $I_{0-0}$/$I_{0-1}$ ratio in the PL spectra of P3HT films signifies a fundamental change in the nature of excitonic coupling and molecular ordering. Specifically, a higher ratio indicates a transition from *H*-aggregate behavior, which is dominated by interchain electronic coupling, to *J*-aggregate behavior, where intrachain excitonic coupling prevails. In *H*-aggregates, polymer chains are closely packed in a face-to-face configuration, leading to strong interchain interactions that make the 0-0 transition partially forbidden and reduce PL intensity. As a result, films with strong *H*-aggregate character typically exhibit higher electrical conductivity because charge carriers can easily hop between chains.

Conversely, when the $I_{0-0}$/$I_{0-1}$ ratio increases, it implies that the polymer chains have become more planar and electronically isolated, favoring intrachain delocalization of excitons characteristic of *J*-aggregates. This configuration enhances the radiative recombination of excitons, resulting in stronger PL emission and longer exciton lifetimes. The increase in this ratio is therefore associated with reduced torsional disorder along the polymer backbone and improved π-conjugation, which enhances optical performance but can limit interchain charge transport.

In the context of P3HT thin films, this means that as the $I_{0-0}$/$I_{0-1}$ ratio increases, excitons are more likely to recombine radiatively within individual chains rather than transferring between chains. While this benefits light-emitting applications, it may reduce the overall mobility in FETs because efficient charge transport depends on interchain connectivity. Therefore, the $I_{0-0}$/$I_{0-1}$ ratio serves as a key indicator of the balance between optical activity and charge transport efficiency, reflecting the competition between intrachain (*J*-type) and interchain (*H*-type) electronic coupling in conjugated polymer systems like P3HT.

**Figure 6:**
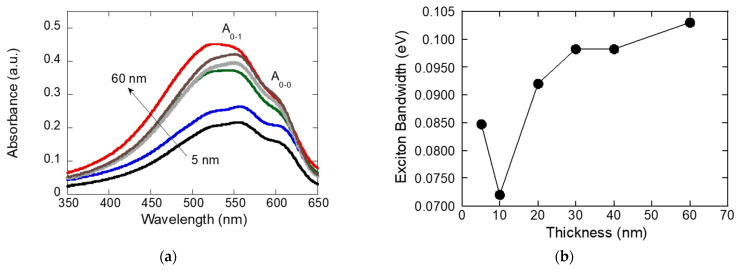
(**a**) Absorbance spectra of P3HT films with different thicknesses (5–60 nm), showing increased absorbance and spectral feature evolution as thickness increases. (**b**) Exciton bandwidth as a function of P3HT film thickness, demonstrating the variation in exciton coupling and molecular packing with film thickness.

We also performed thickness-dependent absorption measurements to correlate the exciton bandwidth (W) with film thickness. The observed variation in exciton bandwidth (*W*) with P3HT film thickness obtained from thickness-dependent absorption plots in Figure 6a reveals critical insights into how molecular ordering and aggregate type influence charge transport. Following the weakly interchain-coupled, modified Franck–Condon (Spano) model [31], we estimated the free-exciton bandwidth W from the vibronic intensity ratio in the absorption spectra. Specifically, the ratio of the 0-0 to 0-1 bands satisfies Equation (3):

(3) $$\frac{A_{0-0}}{A_{0-1}}\approx {\left(\frac{1\;-0.24\;W/\hbar \omega_{0}}{1\;+0.073\;W/\hbar \omega_{0}}\right)}^{2}$$

where $A_{0-0}$ and $A_{0-1}$ are the oscillator strengths of the corresponding vibronic transitions, and $\omega_{0}$ denotes the vibrational mode coupled to the electronic transition ($\hbar \omega_{0}\approx 0.18\;\mathrm{eV}$ for P3HT films). Using this relation, we extracted the exciton bandwidth (W) for each sample; the resulting values are presented in Figure 6b.

As the film thickness increases initially, the exciton bandwidth *W* calculated from the absorbance spectra in Figure 6a decreases, indicating improved molecular ordering and enhanced π–π stacking between polymer chains. The exciton bandwidth *W* reflects the energetic range over which excitons are delocalized within and between chains: a lower *W* suggests that exciton states are more confined due to highly ordered, well-coupled polymer backbones, while a larger *W* typically arises from greater structural disorder or stronger interchain interactions. Therefore, the observed decrease in *W* with increasing thickness at first signifies a progression toward a more ordered and better-stacked P3HT microstructure, which is beneficial for optoelectronic performance.

At very low thicknesses (5–10 nm), the exciton bandwidth *W* decreases from about 0.085 eV to 0.072 eV because the film is dominated by substrate interface effects. In this regime, P3HT chains lie flat against the substrate, enhancing intrachain planarity and suppressing intermolecular interactions. This promotes *J*-aggregate character and thus lowers *W*. As the film grows thicker (10–20 nm), *W* rises sharply to around 0.098 eV. Here, bulk aggregation begins: π–π stacking between chains forms readily, and *H*-aggregate components surge. The stronger interchain excitonic coupling and increased structural disorder in this transitional region drive the rapid increase in *W*. Beyond 30 nm, W continues to climb up to about 0.103 eV as the bulk regime takes over. The diminishing influence of the interface allows bulk *H*-aggregate characteristics to dominate, yielding the highest *W* values. This U-shaped *W* vs. thickness curve reflects the competition between interface-induced *J*-aggregate ordering at low thicknesses and bulk-driven *H*-aggregate aggregation at greater thicknesses. The sharp transition around 10 nm marks the structural crossover from interface control to bulk aggregation, highlighting a critical thickness for tuning P3HT film properties in optoelectronic applications

The exciton bandwidth (*W*) plot as a function of P3HT thickness in Figure 6b offers important insight into the correlation between aggregate type and electrical mobility. Around 10–20 nm thickness, the electrical mobility reaches its maximum, which aligns with a region where exciton bandwidth rises sharply from its minimum value. At these intermediate thicknesses, the film transitions from being dominated by interface-induced *J*-aggregate character to a regime where optimal H-aggregate formation begins. *J*-aggregates are associated with high intrachain order and relatively weak interchain electronic coupling, while *H*-aggregates possess strong interchain π–π stacking that enhances electronic delocalization. Mobility is highest when these two aggregate types are balanced—*J*-aggregates provide extended, orderly charge transport paths along polymer chains, and the emerging *H*-aggregate structures link these paths through efficient interchain hopping. At lower thicknesses, limited *H*-aggregate domains and poor bulk crystallinity restrict mobility, while at greater thicknesses, excessive *H*-aggregate content and increasing structural disorder lead to charge trapping and reduced mobility. Thus, the peak in mobility at 10–20 nm reflects an optimal microstructural balance between *J*-aggregate induced chain ordering and *H*-aggregate-enabled interchain connectivity, producing the most favorable conditions for charge carrier transport. Taken together, the PL spectra and vibronic ratio analysis show that sonicated P3HT films exhibit an optimum thickness range where *H*-aggregation is strongest, leading to maximum exciton quenching. This behavior correlates with electrical measurements where the peak field-effect mobility was observed at a similar thickness, confirming that strong interchain interactions and aggregation drive both reduced PL emission and enhanced charge transport efficiency [32,33].

**Figure 7:**
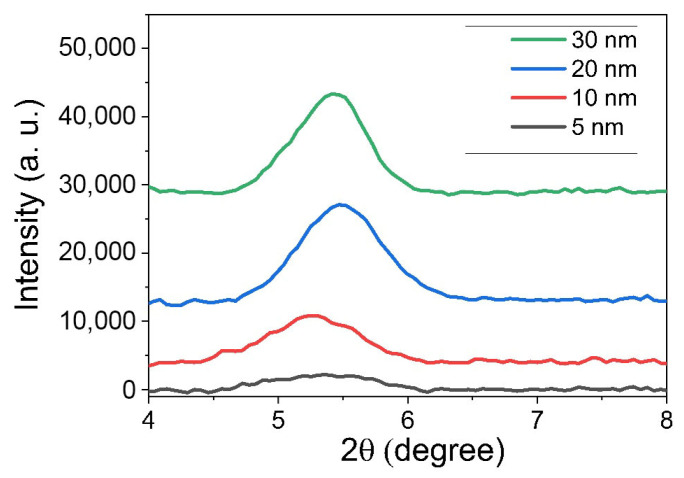
X-ray diffraction (XRD) patterns of P3HT thin films with varying thicknesses (5, 10, 20, and 30 nm).

The XRD pattern in Figure 7 reveals that as the thickness of P3HT increases from 10 nm (red) to 20 nm (green), the intensity and sharpness of the (100) diffraction peak undergo significant changes, corresponding to enhanced crystallinity and more pronounced lamellar ordering. At 5 nm, the XRD peak is weaker and broader, indicating smaller crystalline domains and more amorphous content. As thickness increases from 10 to 20 nm, the peak becomes sharper and much more intense, reflecting improved molecular ordering and the growth of well-aligned crystalline regions.

The thickest films (20 nm and 30 nm) display the most intense and sharp peaks, while thinner films (5 nm, 10 nm) show much weaker signals. As the P3HT film thickness increases from 10 nm (red line) to 20 nm (blue line) and 30 nm (green line), the center of the (100) diffraction peak shifts slightly to a higher 2θ angle (shifting from 5.3° to 5.5°). A shift to a higher diffraction angle corresponds to a decrease in the *d*-spacing (inter-planar distance). In the context of the P3HT (100) peak, this represents a reduction in the lamellar spacing between the polymer backbones separated by alkyl side chains. This reduction in *d*-spacing indicates tighter lamellar packing and more interdigitated alkyl side chains as the film thickness increases towards the optimum. This tighter packing is a signature of enhanced structural order and crystallinity, which supports that the film evolves from a disordered state into a highly ordered, well-packed microstructure at these intermediate thicknesses.

This structural improvement at 20 nm and 30 nm directly correlates with the PL and absorption results: PL measurements show an increase in exciton bandwidth and a slight decrease in the $I_{0-0}$/$I_{0-1}$ ratio, indicating the emergence of *H*-aggregate character and increased interchain coupling. Absorption spectra also exhibit red shifts and vibronic peak narrowing due to better π-π stacking. The XRD plot demonstrates that P3HT films become more crystalline and exhibit stronger, sharper diffraction peaks as the thickness is increased from 5 nm to 30 nm. This is typical for many conjugated polymers like P3HT, where sufficient film thickness is required to achieve optimal crystalline packing and high-quality structural order.

This optimal crystallinity and aggregate balance at around 20 nm facilitate maximized charge transport, as observed by the peak in electrical mobility. The improved ordering provides extended charge percolation pathways, while the balanced aggregate types minimize trapping and promote efficient carrier hop between domains. As the thickness further increases to 30 nm (blue), the XRD peak is sustained, but additional disorder and vertical inhomogeneity begin to limit carrier movement, as also reflected by slightly reduced mobility and broadened PL and absorption features. Thus, the highest mobility at intermediate thickness arises from the interplay of crystallinity, optimal aggregate formation, and structural uniformity evidenced across XRD, PL, and absorption data.

The thickness-dependent electrical and optical measurements reveal a consistent picture of how aggregation governs charge transport in sonicated P3HT. In Figure 4, the four-contact effective mobility exhibits a clear maximum at ~15–16 nm, beyond which mobility decreases and eventually saturates. This optimum thickness coincides with the sharp rise in threshold voltage, reflecting the accumulation of mobile carriers once deep traps are filled. Such non-monotonic transport behavior points to a structural origin, where aggregation and crystallinity are maximized at intermediate thickness but compromised by disorder in thicker films.

The threshold voltage ($V_{T}$) in a P3HT FET varies with the film thickness because increasing the thickness affects the accumulation of charge carriers and the degree of disorder, particularly near the interface between the semiconductor and the gate dielectric. In thin P3HT films, the interface is more strongly coupled to the gate, and any traps or impurities near this region will play a greater role, often requiring a lower threshold voltage for conduction to begin. As the thickness increases beyond a certain value (about 15–16 nm), the threshold voltage rises sharply and saturates at a higher positive value. For a *p*-type semiconductor, a positive threshold voltage means that, even when the gate voltage is zero, there are already sufficient mobile holes present—often due to intrinsic doping, charge trapping, or impurities within the thicker film. This implies that the system tends to form a channel even without significant gate-induced accumulation. As thickness increases up to around 16 nm, mobility rises—likely because the film becomes more continuous and less influenced by interfacial defects, leading to fewer traps and better percolation paths for the charge carriers. Beyond this thickness, mobility begins to decline, likely due to increased bulk disorder and the presence of more trapping sites or grain boundaries within the thicker film. The rapid increase in threshold voltage after this critical thickness suggests increased charge trapping or fixed charges in the bulk of the film, requiring higher gate voltage to turn the device off or to remove excess holes [34].

On the other hand, the non-monotonic thickness dependence of mobility in sonicated P3HT films can be directly correlated with the transient current behavior observed during channel formation in the drop-casting experiment described in the previous study [30]. In the drop-casting process, the drain current initially increased as solvent evaporation promoted polymer aggregation and the formation of interconnected percolation pathways. This current reached a peak at an intermediate stage of solvent evaporation, corresponding to the point where intermolecular ordering and π–π stacking were maximized. As evaporation proceeded further, however, excessive aggregation and chain immobilization disrupted the conductive network, resulting in a gradual decrease in current.

A similar aggregation-controlled behavior is manifested in the solid-state FETs studied here. As the P3HT film thickness increased, the mobility first rose sharply, reaching a maximum at an optimum thickness where molecular ordering and lamellar stacking were most favorable. This peak reflects a structural regime where the polymer chains possess sufficient aggregation to ensure strong interchain coupling, yet remain sufficiently ordered to minimize energetic disorder. Beyond this thickness, however, the mobility decreased, consistent with the formation of excessive *H*-aggregates and disordered grain boundaries that hinder carrier transport. The thickness-dependent mobility trend thus parallels the transient current profile observed during drop-casting, both governed by the same underlying mechanism of dynamic aggregation and structural evolution.

This correlation reveals that the kinetics of aggregation during film formation play a decisive role in defining the final transport properties of conjugated polymer films. The transient current peak in the drop-casting process represents the temporal analog of the optimum thickness in the FET device: both signify the point at which aggregation and ordering are balanced for maximum carrier mobility. Therefore, the solidification dynamics of the polymer solution are directly linked to the steady-state charge transport in the resulting film. Understanding and controlling this dynamic balance between chain aggregation and disorder during film formation provide a powerful strategy to engineer the morphology and optimize charge transport in conjugated polymer-based devices.

## 4. Summary and Conclusions

To summarize, thickness-dependent PL, absorption, and XRD together point to an aggregation-driven structural optimum that maximizes charge transport in sonicated P3HT. With increasing thickness, the ratio drops to a minimum at an intermediate film thickness, indicating strongest *H*-aggregate (interchain) coupling and maximal exciton quenching. This is the thickness where chains are most planar and interchain π–π overlap is best connected laterally—conditions that favor hole transport in the FET channel. The highest mobility occurs at 10–20 nm where the exciton bandwidth sharply rises because this thickness features an optimal mix of *J*-aggregates (which enhance chain ordering) and *H*-aggregates (which strengthen interchain coupling). *J*-aggregates provide good intrachain transport, while *H*-aggregates link these chains for efficient carrier hopping. Mobility drops at lower thickness due to limited *H*-aggregate formation, and at greater thickness due to excessive *H*-aggregate content and increased disorder, which cause more trapping. Thus, mobility peaks when *J*- and *H*-aggregate contributions are balanced. From XRD, the (100) lamellar peak sharpens and intensifies up to this thickness, often with a slight reduction in *d*-spacing, evidencing larger crystalline coherence length and tighter lamellar packing. Beyond the optimum, peak broadening/plateaus indicate added disorder and grain-boundary density. In other words, peak mobility arises when interchain *H*-aggregate order is maximized without incurring thickness-induced disorder, a point identified consistently by the PL minimum ($I_{0-0}$/$I_{0-1}$), the absorption-derived minimum *W*, and the XRD sharpening of the (100) reflection.

This research delves into the profound effects of aggregation on the electronic behavior, suggesting that electrical gating properties can be effectively controlled through the manipulation of aggregate formation, driven by strong polymer chain interactions. Together, the results presented here reveal a delicate balance between film thickness, aggregation, and electrical performance. The coincidence of maximum mobility and increasing threshold voltage near 15–16 nm highlights the role of controlled aggregation in optimizing transport properties. Specifically, sonication-induced *H*-aggregate formation appears to produce an optimal microstructure at intermediate thickness, where extended conjugation and favorable interchain overlap maximize charge transport efficiency before disorder dominates at larger thicknesses. This understanding opens up new possibilities for optimizing the structural arrangement in solution-state P3HT for specific applications. Such insights are particularly relevant in fields like solar cell and sensor technology, where the efficiency of light-induced current generation is a critical factor.

## Data Availability

The data presented in this study are available on request from the corresponding author.

## Supplementary Materials

The following supporting information can be downloaded at: https://www.mdpi.com/article/10.3390/ma19020279/s1, Figure S1. AFM height images of (a) pristine P3HT and (b) sonicated P3HT thin films. The sonicated film displays enhanced textural features and domain formation, consistent with the increased aggregation and crystallinity indicated by UV-Vis and XRD analysis; Figure S2. Sheet conductance as a function of gate voltage (*V_G_*) for sonicated P3HT films with thick-nesses of (a) 7 nm and (b) 150 nm. The linear fits (red lines) to the experimental data (black circles) in the linear regime effective four-contact field-effect mobilities of 3.1 × 10^−4^ cm^2^/Vs (7 nm) and 3.4 × 10^−3^ cm^2^/Vs (150 nm), respectively. The excellent linearity (R^2^ ~ 0.99) confirms reliable extraction of transport parameters well beyond the optimum thickness range.
