# Supplementary material for: Aggregation-Tuned Charge Transport and Threshold Voltage Modulation in Poly(3-hexylthiophene) Field-Effect Transistors
Source: Materials (Basel). 2026 Jan 9;19(2):279. doi: 10.3390/ma19020279 (PMC12842914; doi:10.3390/ma19020279)
Supplement: Supplementary file 1 [file materials-19-00279-s001.zip › materials-3984282-supplementary.pdf]

Supporting Information

Aggregation-Tuned Charge Transport and Threshold Voltage Modulation in Poly(3-Hexylthiophene) Field-Effect Transistors

Byoungnam Park

Department of Materials Science and Engineering, Hongik University, 72-1, Sangsu-dong, Mapo-gu, Seoul 04066, Republic of Korea; metalpbn@hongik.ac.kr

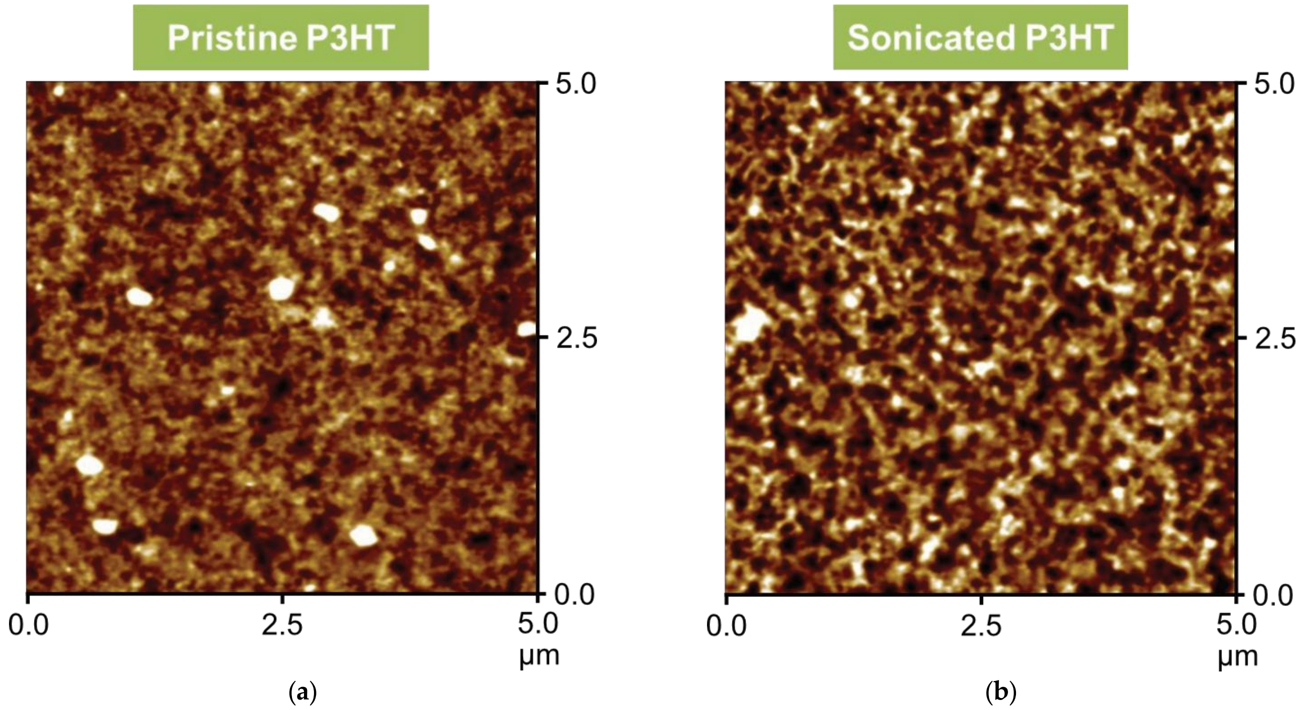

Figure S1. AFM height images of (**a**) pristine P3HT and (**b**) sonicated P3HT thin films. The sonicated film displays enhanced textural features and domain formation, consistent with the increased aggregation and crystallinity indicated by UV-Vis and XRD analysis.

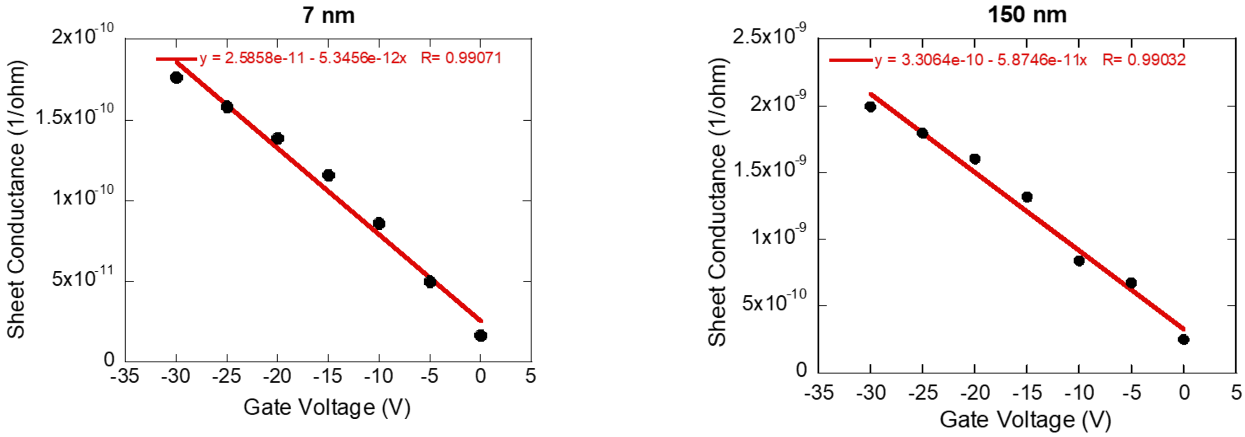

(**a**) (**b**)

Figure S2. Sheet conductance as a function of gate voltage (V_G) for sonicated P3HT films with thicknesses of (**a**) 7 nm and (**b**) 150 nm. The linear fits (red lines) to the experimental data (black circles) in the linear regime effective four-contact field-effect mobilities of 3.1×10^{-4} cm^2/Vs (7 nm) and 3.4×10^{-3} cm^2/Vs (150 nm), respectively. The excellent linearity ($R^2 \sim 0.99$) confirms reliable extraction of transport parameters well beyond the optimum thickness range.
